# Factors associated with negative energy balance in periparturient dairy cows raised under tropical climate of Thailand—A mini-review

**DOI:** 10.5455/javar.2021.h526

**Published:** 2021-09-01

**Authors:** Supawit Triwutanon, Theera Rukkwamsuk

**Affiliations:** Department of Large Animal and Wildlife Clinical Sciences, Faculty of Veterinary Medicine, Kasetsart University, Kamphaeng Saen, Nakhon Pathom, Thailand

**Keywords:** Dairy cow, negative energy balance, small-holder farm, tropical climate

## Abstract

This review attempted to explain factors associated with negative energy balance (NEB) occurring during the periparturient period in dairy cows raised under tropical climatic conditions. The NEB has long been proven as an inevitable event in periparturient dairy cows. This condition had negative effects on the overall performances of dairy cows, including milk production, reproduction, and health condition. Therefore, periparturient management to overcome the NEB problem is vital for optimizing profit in dairy farming. In most tropical countries such as Thailand, dairy cows have been predominantly kept by small-holder farmers. Consequently, baseline milk yields, feed availability, feeding management, and general farming practices are different from typical commercial dairy farming. Heat stress also plays a crucial role in NEB conditions, and elevated temperature-humidity indexes above-normal conditions are recorded throughout the year. These factors influence the NEB in tropical dairy cows, which could result in different outcomes and consequences. Understanding the affecting components of NEB in dairy cows would help alleviate the severity of the NEB and its consequences, optimizing the dairy cow’s performance.

## Introduction

The period from late pregnancy to early lactation in dairy cows is crucially essential for milk production, health condition, and reproductive performance [1]. During this phase, cows usually face various physiological changes ranging from reduced milk production, rapid fetal growth, and decreased dry matter intake (DMI) to rapidly increased milk production and increased nutrient demands [2,3]. As a consequence, dairy cows have to adapt and compensate for these changes. Therefore, blood biochemical parameters, namely, glucose, non-esterified fatty acids (NEFA), β-hydroxybutyrate (BHBA), triacylglycerol (TAG), cholesterol, total protein, albumin, urea nitrogen, bilirubin, and some metabolic hormones such as growth hormone (GH), insulin and insulin-like growth factors (IGF) and its binding proteins are altered [4–7]. The alteration of these blood biochemical parameters and plasma metabolic hormones cannot balance the energy demand during the transition period. In that case, cows will enter the negative energy balance (NEB) status, directly or indirectly affecting their health, milk production, and reproduction [3,8]. To gain profits from dairy cows, it is essential to properly manage housing, feeding, health, and reproduction to help cows go through this period without any adverse consequences. 

Various factors are affecting NEB condition in dairy cows. For energy requirements, a genetic factor predominantly related to milk production is an influential parameter [9], together with environmental and management factors such as temperature, humidity, or housing system [10]. For energy intake, feeding management and feed ingredients for proper animal intake and requirement amounts are the most important factors [11]. All of those factors are highly dependent on geographic differences in each region. For example, each geographic area has its conventional forage and concentrate ingredient availability, which directly influences nutritional management. The temperature and humidity are also be considered important factors because they have directly affected the DMI and nutrient requirement of dairy cattle [12]. 

Up until this date, most studies about NEB in dairy cows have been done in temperate areas, which are suitable and convenient for dairy cattle farming in Europe and North America [13]. In tropical zones, especially Thailand, which have a reasonable number of dairy farms, data of NEB and its related factors, including changes of blood biochemical parameter profiles, hormonal alteration, changes of liver fat content, fluctuation of milk composition, and milk somatic cell counts throughout the periparturient period of dairy cows are not yet widely reviewed.

### Physiological changes and negative energy balance

The periparturient or transition period is the phase that influences dairy cows′ production system throughout the cycle [14]. In this period, DMI is decreased by 30% after parturition, but glucose demands may increase 2 to 5 times as high as the non-lactating phase to match milk production after parturition [15]. These physiological changes result in the NEB problem of periparturient dairy cows. Both metabolic hormones and their receptors could be altered over this critical period to match the physiological changes, particularly GH, insulin, IGF, and glucose concentrations. Metabolic hormones and their receptors are affected. Besides, various blood biochemical parameters are affected, such as glucose, NEFA, and BHBA.

In response to high blood glucose demands from NEB, alteration of insulin-glucagon ratio, and other metabolic hormones trigger the activation of hormone-sensitive lipase to increase lipolytic rate in adipose tissue [16]. An increase in lipolysis occurs to release NEFA and glycerol into blood circulation, leading to hyperlipidemia or high NEFA in the serum of post-parturient dairy cows. Both substances were uptaken mainly in the liver; glycerol enters the gluconeogenesis pathway resulting in serum glucose or recombine with NEFA. NEFA is oxidized in the *β*-oxidation reaction to produce 2-carbon fatty acids and converted to acetyl coenzyme-A before combining with oxaloacetate to enter the tricarboxylic acid cycle. The use of oxaloacetate in this pathway competes with the gluconeogenesis pathway. Suppose oxaloacetate is insufficient or limited from the dramatical use in the gluconeogenesis pathway together with decreasing DMI; in this case, acetyl Co-A will be converted to ketone bodies, which in high serum concentrations can cause various adverse effects on the production and animal health [13,17]. NEFA can recombine with glycerol and deposit in hepatocytes, resulting in the fatty liver or hepatic lipidosis, which leads to impairment of liver functions [13]. There is another pathway to secrete TAGs from the liver into blood circulation by forming TAGs, and other liver synthesis components such as apo-protein and phospholipid resulting in very-low-density lipoprotein, but the rate of synthesis is low in the ruminant compared to other species [14]. 

The hormonal alteration also occurs. IGF-I is a peptide hormone that plays a crucial role in dairy cattle production. The IGF-I was produced mainly by the liver and bound with insulin-like growth factor binding protein to release into blood circulation. Several factors affecting plasma IGF-I level are milk yield, nutritional status, age, and liver function. Previous studies showed that plasma IGF-I levels are positively related to growth [18], reproduction [19,20], and milk production in dairy cows [21]. 

### Consequences of negative energy balance on production

When NEB occurs, several consequences may happen, such as hyperketonemia, hyperlipidemia, hepatic lipidosis, and reduced IGF-I synthesis, which result in negative effects on milk production, reproductive performance, and overall health of animals. To a high degree, hepatic lipidosis reduces general liver functions, increases the apoptosis rate of hepatocytes, and induced endoplasmic reticulum stress [22–24]. Interestingly, periparturient dairy cows show increased lipid oxidation, mitochondria function, and autophagy activity at the mild degree of hepatic lipidosis. These metabolism changes show metabolic adaptation of dairy cows during the peri parturition stage [25,26]. For related conditions, hyperketonemia has various effects depending on the degree of concentrations, such as reduced DMI or decreased immune function [2,4], but in severe cases, it can lead to the death of a cow.

Effect on overall performance, including milk, reproduction, and animal health, was reported. For milk production, the direct effect of NEB on the reduction of milk yield, both short and long term, was reported together with an indirect effect on udder health and immune function [27–29]. The relation of NEB to health problems such as lameness, abomasal displacement, metabolic disease, and reproductive disease, including metritis and retained placenta [7,30], are reported. *In vitro* chemotactic impairment of leukocytes treated with ketone body and increased culling rate related to NEB of periparturient dairy cows are also published [28,31].

Dairy cows with NEB have decreased reproductive performance by alteration of DMI to reduce nutritional activation of the reproductive cycle [9,32,33]. 

### Factors associated with negative energy balance

#### Milk yield

The degree of severity in NEB depends on various factors that affect energy balance, mainly milk production, and nutritional management. Therefore, the difference in milk production between herds or between areas should be concerned when discussing NEB conditions. Data from 2015 in Thailand, there were 509,524 dairy cattle, of which 286,296 were milking cows, kept by 16,248 dairy cattle farms. Most farms are located in the central and western regions [34]. The major breed is Holstein Friesian crossbred with the tropical cows, of which the Holstein bloodline (*Bos taurus*) was over 87.5%. Other breeds such as Jersey, Brow-Swiss, and tropical dairy milking breeds were also found without quantitative data. The average milking cow per farm was 17.62 cows per farm [34], which is lower than other developed dairy industrial countries such as the USA, Israel, Australia, and other EU countries [35]. According to the dairy farming category under Thai conditions, 39.19% and 15.5% of farms had milking cows less than 12 and 6 cows per farm, respectively [34]. 

From overall data, we could describe that dairy cattle farms in Thailand were mainly small-holder farms. In this type of farm, records and data collection for milk production, reproductive events, and other information are still lacking. Some milk information were reported only by the farms under the Dairy Farming Promotion Organization of Thailand. Average daily milk production per cow was 12.02 ± 0.75, 9.59 ± 0.93, 11.00 ± 0.76 and 10.75 ± 0.98 kg, and the average number of milking cows per farm were 18.74 ± 1.93, 9.75 ± 2.41, 12.13 ± 1.96, and 21.77 ± 2.54 in the central, northern, northeast, and southern parts of Thailand, respectively [34]. The overall country data of dairy cow production were also reported by Aiumlamai [36]; 3,945 ± 1,537 kg of milk/lactation and 324 ± 97 days of lactation length. Milk production of dairy cows in Thailand is lower than in other milk-producing countries [10], but the NEB problem still occurred. Energy used for milk production is the main requirement during the postpartum period. With lower production, the elevation of serum BHBA, NEFA, and liver TAG had been observed, indicating subclinical ketosis of dairy cows raised in Thailand [37]. Therefore, other factors affecting NEB, such as feed intake, nutrient availability, postpartum diseases, or heat stress, should be considered when evaluating the NEB condition in peripartum cows. 

#### Feedstuff availability

Thailand is a country in a tropical area with adequate rainfall and good irrigation management [38]; therefore, plenty of agricultural plants can be cultivated all year round [39]. However, Thai-traditional dairy farming, which mainly consisted of a small-holder farming system. Data from the Thai government showed that 33.74% and 63.70% of dairy farmers had less than 20 heads and 20–100 heads of total dairy cattle within a herd, respectively [40]. The usage of agricultural lands in 2020 was 92.32% as plant cultivation, resulting in limited land for ruminant-forage production [41]. Therefore, the main forage and fiber sources for dairy cows in Thailand are mainly originated from agricultural by-products and left-over of other cultivated crops [42], e.g., rice straw, corn husk, and post harvested corn silage (without corn stalk) from sweet corn and baby corn. Nutritional values and the price of available forage and non-forage fiber sources for dairy cattle in Thailand are presented in Table 1 [43].

**Table 1.** The nutritional value and price of available forage and non-forage fiber sources for dairy cattle in Thailand (Price calculated based on currency exchange, 1 USD = 33 BTH).

Type of forage		Dry matter (%)	Crude Protein (%)	TDN (%)	Ether extract (%)	Price per ton of dry basis [THB (USD)]	
Typical forage	Rice straw *(Oryza sativa Linn.)*	90.16	5.23	57.69	0.07		2,773 (84.0)
	Napier Glass *(Pennisetum purpureum)*	23.86	8.91	56.87	1.63		5,029 (152.4)
	Corn silage *(Zea mays* Linn.)	25.63	8.34	59.06	0.85		5,462 (165.5)
	Corn husk *(Zea mays L. var. saccharata)*	22.28	6.18	59.07	1.12		4,488 (136.0)
High-quality forage	Alfafa hay *(Medicago sativa)*	90.07	18	62	-		19,984 (605.6)
	Alfafa silage	50.08	18	62	-		27,733 (840.4)
	Corn silage (*Zea mays* subsp. *Mays*)	30.45	7.40	70.38	3.06		6,568 (199.0)
None-forage fiber source	Cassava pulp	14.47	2.35	72.1	0.14		5,528 (167.5)
	Cassava-ethanol by product	22.72	12.00	70.7	1.90		3,521 (106.7)
	Soybean pulp	19.26	31.51	80.36	8.88		11,422 (346.1)
	Palm oil sludge	23.49	15.37	9.86	43.4		2,128 (64.5)

Other agricultural industries, for example, cassava starch and palm oil, also play a vital role in the Thai economy. There are plenty of by-products available for ruminant feedstuff as non-forage fiber sources with these types of industry. Cassava pulps, cassava-ethanol by-product, and palm-oil sludge are non-forage fiber sources that are commonly used in Thailand because of their relatively low price. Not only the feedstuff, soybean pulp from the soybean milk industry, and brewer’s grain from the liquor industry with higher nutritional value are also used as ruminant feedstuff. However, the latter is not extensively used mainly due to the higher costs than other by-products. These by-products from other agriculture industries can reduce the feed cost in a dairy farm. Still, it could come with another limitation of their use: uncontrollable and varied moisture between different lots of by-products. With a lack of moisture evaluation in the feed ingredient at the farm level, farmers might underfeed their dairy cattle in dry matter basis, especially in high-variable moisture feed. The overuse of non-forage fiber in dairy cows also reduces the ratio of effective neutral detergent fiber leading to subacute ruminal acidosis, which is considered an important problem as its prevalence has been reported to be 30% to 42% [44,45]. Various nutritional limitations and diseases followed by metabolic disorders, especially in transitional dairy cows, enhance greater chances of NEB condition [46].

The actual forage of dairy cattle widely cultivated in Thailand is Napier grass (*Pennisetum purpureum*) and its related species because of higher yields per area than the other forage types. In the non-water irrigating area, annual cumulative yields were 26.1 to 31.3 tons/ha/year as a dry matter basis for Napier grass cultivars [47]. Other forages, such as Ruzi and Dwarf Napier produce only 18.44 to 20.84 tons/ha/year in similar conditions [48]. 

Other better quality, temperate legume forage, for example, alfalfa, is not commonly found due to uneconomical cultivation and low production per area in the humid-tropical area. Whole corn (*Zea mays* subsp. *Mays*) silage is typical temperate forage that can be cultivated in Thailand. This type of cultivation can be found only in some areas because of water availability for cultivation. The use of corn silage is still limited because of the inability to balance the demand and supply of corn silage to dairy farms, and also it is more expensive than other by-product forage. The prices of typical corn silage were 146.92 and 229.56 USD per ton of dry matter basis in the bunker silo and the plastic bag, respectively, which were higher than the prices of other forage and by-products as presented in Table 1.

The inability of small-holder dairy farms, which mainly use workforce to manage the forage, is another limitation in using and transporting some forage. Most Thai dairy farmers still used the cut-an-carry method to harvest forage. Therefore, the small-scale machines were used to reduce particle length in the Napier related to grass; however, a complete harvest using an advanced machine was rarely seen. For forage preservation, some types of feedstuff had to be stored as fermented feed. Due to the low amount of daily feed consumption in relation to low cow numbers and lack of technology to manage fermented feed, inadequate forage and failure to preserve good quality forage during the dry season could occur. Both feed quality and quantity problems, under-feeding, low DMI, and insufficient nutrient consumption possibly lead to NEB in postpartum dairy cows. On the other hand, high moisture feed that limits forage intake and failure to manage feed particle sizes also increased the risk of ruminal acidosis that had a negative effect on energy balance [36].

The concentrate is another factor that influent nutritional management of dairy cow. The price of some common feedstuff compared in Table 2 was 2.3 and 1.2 times higher for corn and soybean meal in Thailand than in the USA, respectively [49–51]. Another factor to concern is that a small-holder farm in Thailand cannot produce their concentrate. As a result, they use commercial feed. By the law of the Thai government, the producers of concentrate feed for animals have to describe nutritional values, including moisture, protein, fiber, and ash; that unable to calculated energy value, for example, total digestibility nutrient (TDN) by the farmer.

A study about the cost of milk production in Thailand showed that 49.36% was for the cost of cattle feed. Thus, it was divided into 39.04% and 10.32% of concentrate and forage, respectively. Concentrates shared an economically high cost in Thai milk production [52]. Factors including the high price of concentrate feed, lack of proper feeding management in small-holder dairy farms, and inability to formulate on-farm concentrates should be considered an NEB-related factor in Thailand. In Thai small-holder farms, mismanagement of the concentrate diet resulted in over-condition prepartum or under-condition early postpartum, leading the cow to enter into NEB [37,46]. 

#### Heat stress

In the past 40 years, genetic improvement of dairy cattle has been made successfully, especially the Holstein-Fresian breed. Increasing in milk production in the USA dairy industry was four times greater in 1995 than in 1940, but it also changed in the thermal regulation of dairy cattle breed [10]. Higher producing dairy cows have lesser heat tolerance and higher susceptibility to heat stress problems. In general, dairy cows are considered in their comfort zone at a temperature of 6°C to 18°C. In a tropical area with a high humidity variation, the temperature-humidity index (THI) value is calculated based on environmental temperature and humidity. The THI value should be used as an indicator to predict heat stress problems in dairy cows [53].

**Table 2.** Comparison of the average price of corn and soybean meal as animal feed between 2007-2016 in USA and Thailand (Price calculated based on currency exchange (1USD = 33BTH).

Type of concentrate	Price per ton in USA (USD)	Price per ton in Thailand (USD)	Price comparison (Percentage of Thai/USA price)
Corn	109.15	252.12	230.9%
Soybean meal	362.00	434.84	120.12%

Heat stress has various negative effects on dairy cows ranging from reduced milk production to impaired health status [54,55]. In adaptation to increased environmental temperature, the basal metabolic rates decrease as compared to normal temperature. In contrast, the DMI is decreased greater than the metabolic rate, resulting in a higher risk of the dairy cow entering a preliminary NEB stage [56].

Heat stress and consequences can lead to higher chances for dairy cows to enter the NEB stage. Not only directly resulting from nutritional factors, the effect of heat stress on other factors such as impairment of various hormones in the GH-IGF axis [12], behaviors change [57], and immunosuppression [58] are also indirect causes of NEB condition. 

**Figure 1.**
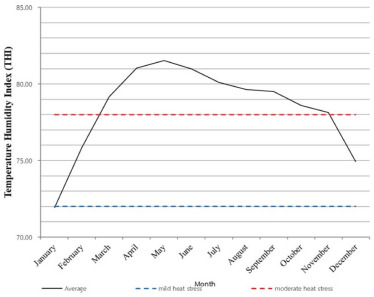
The average monthly temperature-humidity index of Thailand in general and divided by region in 2014.

The guidance for the management of heat stress problems based on THI is set. The THI levels for mild, severe, and very severe heat stress are 72–78, 78–89, and 89–98, respectively [59]. The THI of Thailand is demonstrated in Figure 1 [60,61]. If we consider the average THI, cows in a year were in normal, mild, and moderate heat stress conditions for 1, 2, and 10 months, respectively. In Thailand’s central and northeastern regions, the maximum temperature during the summer period usually exceeds 40°C, which can cause dairy cows to enter severe heat stress conditions. The THI in the northern and northeastern regions is lower than the country’s THI average [62]. The severity of heat stress classified by average THI was within mild stress from October to March and severe stress from April to September.

The relation between days open, the month of calving, and THI are also reported in the same study [62]. The calving month was notably in September, which was corresponded well with successful breeding from December to January when the THI was low. Dairy cows calving in other months, which are hotter at the time of rebreeding period, had longer days open than cows that calved in October. 

### Negative energy balance study in Thailand

Studies of NEB in dairy cows include various data and multiple sample collections, which were inconvenient for fully convincing small-holder farms. The overall management of Thai dairy farms was similar to other developing countries, a small-scale farming system with incomplete basic data collection, i.e., identification number, age, date of calving, artificial insemination date, etc., of milking cows. More advanced data and information, such as DMI, daily milk yield, health problems, previous body condition score (BCS), and other reproductive indices, are also unable to collect. Sample collection issues such as blood collection and liver biopsy are also being considered as invasive methods for the farmers′ perception due to a lack of understanding of veterinary procedures. For all of these reasons, most research and studies were done in commercial or research farms, represented in very small numbers in Thailand, instead of typical small-holding farms. 

There are several studies about NEB and related hormonal and biochemical parameters of dairy cows in Thailand. Variation of dairy farms that were studied ranges from the large-scale commercial to the small-holder farms, and milk production ranges from 14 to 40 kg/day.

The dynamic change of BCS at transition period, which is directly related to NEB condition, was studied. The dramatically decreased at 2 weeks prepartum and gradually increased body weights at 3 weeks postpartum of dairy cows were reported [62]. The BCS in prepartum cows was reported [3,8,63,64], lower than the expected BCS of 3.5. The loss of BCS postpartum was reported in various studies ranging from 0.19 to 0.49 units (1 week prepartum compared to 4 weeks postpartum) in approximately 15 kg/day of milk production in the early lactation period. In comparison with Bernabucci et al. [65] and Bruckental et al. [66], the loss of BCS, around 0.7–1.0 units in postpartum dairy cows produced 31.15 kg/day, are much higher than the loss in BCS studied in Thailand.

Energy balance has been reported by Suadsong et al. [67] in a group of cows with 12.6 ± 0.2 kg/day milk production (during 22 weeks postpartum). Nadir of NEB, days to the nadir of NEB, and days to an equilibrium of energy were −3.7 ± 0.4 Mcal/day, 20.0 ± 2.3, and 53.0 ± 4.1 days, respectively. Compared with −5.06 Mcal/day, 9–10 days and 38 days of the nadir of NEB, days to nadir, and days to equilibrium in the temperate area [68]. The severity of NEB is less but prolonged in Thai studies than in other geographic areas, possibly due to lower milk production, and the inability to provide good nutritional management to the correction of energy balance postpartum.

Blood biochemical parameters of periparturient dairy cows in Thailand were reported in various studies. Dynamic changes of serum glucose were reported with a slight increase during the pre-parturition period, a rise at calving, and a decrease immediately after calving without significant differences [62]. In contrast to other within-country studies, a significant decrease in serum glucose at 2 weeks after calving, compared to 2 weeks before the expected calving date, was observed [63,69]. Levels of serum glucose were reported ranging from 26.17 ± 1.47 to 68.6 ± 7.7 mg/dl [64,69] and 26% lower at 2 weeks after calving compares to 2 weeks before expected calving [63]. Moreover, 12%–13% of cows had more than 40 mg/dl of serum glucose postpartum [36]. Decreasing serum glucose postpartum also were reported in other geographic areas, but the level of serum glucose tends to be lower in Thailand compared to greater than 48 mg/dl in other studies [68,70,71].

Serum NEFA was the most studied blood biochemical parameter in Thailand; levels of serum NEFA were reported with 0.56 ± 0.04 mEq/l, 0.58 ± 0.05 mEq/l, and 0.48 ± 0.04 mEq/l at one week prepartum and 2 to 4 weeks postpartum without significant differences by times [3]. On the other hand, changing serum NEFA by time with the highest concentrations observed at 1 week after calving and decreasing after calving throughout 10 weeks postpartum were reported [62]. Chankrachang and Hongyantrachai [69] showed that serum NEFA concentrations at 2 weeks pre-and postpartum were 0.398 ± 0.035 and 0.475 ± 0.049 mEq/l, respectively significant changes. Dynamic of serum NEFA also reported by Suadsong et al. [67], concentrations were significantly decreased from 1 to 6 weeks postpartum and remained unchanged between 6 to 12 weeks with an average of 0.133 ± 0.007 mEq/l throughout 12 weeks. Rukkwamsuk and Panneum [63] also reported other elevated serum NEFA, and the percentage of increase was 132% (2 weeks postpartum compared with 2 weeks prepartum concentrations). 

Serum NEFA concentration in most studies, except from Suadsong et al. [67], is classified as high as a subclinical ketotic group (0.35 ± 0.01-0.37 ± 0.01 mmol/l) from another study at the different geographic area. As compared to the studies in Thailand, a variation of serum NEFA concentrations was also found in the temperate area [72,73] caused by different milk production and nutritional management between each study. However, significant serum NEFA was reported in many studies in Thailand; therefore, increased lipolytic activity leading to hyperketonemia and hepatic lipidosis should not be ignored. 

A Significant change of serum TAG concentrations was reported at 2 weeks pre-and postpartum [8] with an average concentration in a period of 2 weeks before the expected calving date to 4 weeks postpartum was 17.07 ± 1.21 mg/dl [63]. 

Serum BHBA concentration was reported by Whitaker et al. [74], with average concentrations of 0.8 ± 0.6, 0.9 ± 0.6, and 0.8 ± 0.5 mmol/l for pre-calving, 1 month, and 2 to 3 months post-calving, respectively. The percentage of serum BHBA elevation above baseline value was 9% of the postpartum cows (> 1.2 mmol/l) [36]. Whitaker et al. [74] studied serum BHBA concentrations in sub-tropical and tropical countries in various regions, including Asia, Europe, Northcentral, and South America. A marked decrease in BCS and weight loss after calving were reported in Thailand, and Sri Langa, with one-third of dairy cows in Thailand, had elevated serum BHBA pre-calving (> 0.6 mmol/l), 1 month, and 2 to 3 months post-calving (> 1.0 mmol/l).

In relation to Whitaker et al. [74], the percentage of elevation in serum BHBA above baseline value were reported with 26% (> 0.6 mmol/l) prepartum [4], 31% (> 1.0 mmol/l) [28], and 18%–25% (> 1.2 mmol/l) postpartum [13,75] from other geographic areas, but higher compared to within the country study of Aiumlamai [36]. 

Rukkwamsuk et al. [76] reported a significant difference in serum NEFA and BHBA in dairy cows kept in the evaporative cooling system. Serum NEFA were 0.25 ± 0.06, 0.91 ± 0.12, and 0.73 ± 0.18 mmol/l at 2 weeks before expected calving, 2 and 4 weeks after calving, respectively. Serum BHBA were 0.51 ± 0.05, 1.16 ± 0.21, and 0.90 ± 0.17 mmol/l at 2 weeks before expected calving, 2 and 4 weeks after calving, respectively. Average serum concentrations of NEFA at 2 and 4 weeks after calving and BHBA at 2 weeks after calving were higher than the cut-off for sub-clinical ketosis. Even though elevated serum NEFA and BHBA postpartum were reported, average milk production was 29.1 ± 5.3 kg/cows/d in 30 days postpartum, which was much higher than other studies in Thailand. Due to variation in milk production and evaporative cooling system housing, this study might not represent the typical small-holding dairy farming in Thailand. 

The serum hormone levels were reported by Kaewlamun [62], average 12 weeks postpartum of serum concentration of IGF-I, and cortisol concentrations were approximately 40–100 ng/ml and 40–17 mmol/l, respectively, for dairy cows with average milk production of 15 kg/day at the peak of lactation. There were effects of time postpartum on both blood biochemical parameter concentrations. Before calving, increasing serum cortisol concentrations before calving, declining until 2 weeks postpartum, and remaining stable throughout 12 weeks of the study period were reported. Cortisol remained relatively stable from 2 weeks to the end of the study. Data on serum insulin and IGF-I from cows with an average of 39 ± 7.2 kg/day of milk production were reported in the same study. Serum concentrations of both insulin and IGF-I increased slightly during the 1st month of the dry period and then drastically decline at calving. The lowest blood concentrations of insulin were observed at 2 weeks postpartum. After that, IGF-I increased gradually throughout the rest of the study. Insulin and IGF-I’s serum concentration ranged from approximately 10–25 pmol/l and 100–350 ng/ml, respectively.

Liver TAGs in the cows kept in the evaporative cooling system were reported by Rukkwamsuk et al. [76]. Average concentrations of TAG at 1, 2, 3, and 4 weeks after parturition were 24.3 ± 1.0, 61.4 ± 6.3, 61.1 ± 6.4, and 57.3 ± 6.3 mg/g wet weight of liver, respectively. The prevalence of fatty liver classified by more than 50 mg of TAG in 1 g of the wet weight of liver tissue was 68.4%. Liver TAG concentration was related to another study [6] in the temperate area. This study was conducted in the good management commercial farm with milk production (31 ± 4 kg/d during the first 4 weeks of lactation) and nutritional management similar to commercial farms in other countries. 

Effects of NEB on dairy cow production were also reported [8]. The study showed sub-optimum milk yields, delayed the first estrus after calving, and low pregnancy rates in a group of suspected NEB cows due to significantly reduced serum glucose and increased NEFA at 2 weeks postpartum. Average days from calving to first estrus were 72 ± 65 days, and pregnancy rates in the group studied were 28.6%, 11.1%, and 12.0% for first, second, and third artificial insemination, respectively. Milk production was 15.80 ± 4.10 kg/d during 4 weeks postpartum without a peak of yield between 7 to 9 weeks of lactation.

The study of Chankrachang and Hongyantrachai [69] also reported that days to the first service, days open, services per conception, and first service conception rate were 83.86 ± 4.11 days, 106.76 ± 5.66 days, 1.65 ± 0.14 times, and 47.62%, respectively, which are considered suboptimal for the expected reproductive parameter in a group of cows that are suspected of having NEB.

## Conclusions

A small-holder dairy farm in humid tropical areas was the major farming type in Thailand. Factors related to this type of farming system, including nutritional factors, farm management, heat stress, and feed availability, had unique influences on the NEB condition of dairy cows. Many studies showed that low-producing dairy cows under this farming system demonstrated NEB-related conditions, even at severe degrees in some cows. When evaluating the NEB problem in tropical small-holder farms, nutritional management failures and heat stress should not be overlooked. The small-holder dairy farms are also widely found in other humid tropical climates in South and Southeast Asia. Thus, the pattern of NEB and alteration of biological parameters in Thailand could be used as a model for evaluating NEB conditions in these geographic conditions. 

## List of abbreviations

BHBA: β-hydroxybutyrate, BCS: Body condition score, DMI: Dry matter intake, GH: Growth hormone, IGF: Insulin-like growth factor, NEB: Negative energy balance, NEFA: Non-esterified fatty acid, TDN: Total digestibility nutrient, TAG: Triacylglycerol.
